# Rho kinase-dependent apical constriction counteracts M-phase apical expansion to enable mouse neural tube closure

**DOI:** 10.1242/jcs.230300

**Published:** 2019-07-01

**Authors:** Max B. Butler, Nina E. Short, Eirini Maniou, Paula Alexandre, Nicholas D. E. Greene, Andrew J. Copp, Gabriel L. Galea

**Affiliations:** 1 Developmental Biology and Cancer, UCL GOS Institute of Child Health, London WC1N 1EH, UK; 2 Comparative Bioveterinary Sciences, Royal Veterinary College, London NW1 0TU, UK

**Keywords:** Rock, Posterior neuropore, Apical constriction, Interkinetic nuclear migration, F-actin, Biomechanics

## Abstract

Cellular generation of mechanical forces required to close the presumptive spinal neural tube, the ‘posterior neuropore’ (PNP), involves interkinetic nuclear migration (INM) and apical constriction. Both processes change the apical surface area of neuroepithelial cells, but how they are biomechanically integrated is unknown. Rho kinase (Rock; herein referring to both ROCK1 and ROCK2) inhibition in mouse whole embryo culture progressively widens the PNP. PNP widening is not caused by increased mechanical tension opposing closure, as evidenced by diminished recoil following laser ablation. Rather, Rock inhibition diminishes neuroepithelial apical constriction, producing increased apical areas in neuroepithelial cells despite diminished tension. Neuroepithelial apices are also dynamically related to INM progression, with the smallest dimensions achieved in cells positive for the pan-M phase marker Rb phosphorylated at S780 (pRB-S780). A brief (2 h) Rock inhibition selectively increases the apical area of pRB-S780-positive cells, but not pre-anaphase cells positive for phosphorylated histone 3 (pHH3^+^). Longer inhibition (8 h, more than one cell cycle) increases apical areas in pHH3^+^ cells, suggesting cell cycle-dependent accumulation of cells with larger apical surfaces during PNP widening. Consequently, arresting cell cycle progression with hydroxyurea prevents PNP widening following Rock inhibition. Thus, Rock-dependent apical constriction compensates for the PNP-widening effects of INM to enable progression of closure.

This article has an associated First Person interview with the first authors of the paper.

## Abstract (precis)

**Highlighted Article:** Biomechanical integration of apical constriction and interkinetic nuclear migration in the pseudostratified epithelium of the mammalian spinal neural tube requires the actomyosin regulator Rock.

## INTRODUCTION

Abnormalities in embryonic cellular biomechanics are increasingly recognised as underlying congenital structural malformations in organ systems, including the heart (Hoog et al., 2018), eye (Hosseini et al., 2014; Oltean et al., 2016), joints (Singh et al., 2018) and central nervous system (Galea et al., 2017, 2018). Mechanical forces must be generated to change the shape of embryonic structures into the presumptive organs. These forces may be generated non-cell-autonomously, such as during osmotic swelling of the lumen of the closed neural tube, the embryonic precursor of the brain and spinal cord (Desmond and Jacobson, 1977). Morphogenetic forces are also cell-autonomously generated through conserved mechanisms that alter the shape of cells and, collectively, tissues (Pearl et al., 2017).

Probably the best studied force-generating mechanism is apical constriction of epithelial cells, which requires recruitment of non-muscle myosin motor proteins, such as myosin-II, onto the apical F-actin cytoskeleton. Apical myosin recruitment is promoted by the activity of Rho-associated kinase (Rock; herein referring to both ROCK1 and ROCK2 for mammalian systems) (Das et al., 2014; Mason et al., 2013; Sai et al., 2014). In *Drosophila* and non-mammalian vertebrates, apical constriction proceeds in an asynchronous ratchet-like pulsatile manner, producing wedge-shaped cells with narrowed apical and widened basolateral domains (Christodoulou and Skourides, 2015; Martin et al., 2009). When coordinated across an epithelium, this causes tissue bending (Nishimura et al., 2012).

Although apical constriction has been extensively studied in columnar and cuboidal epithelia, its regulation and function in highly complex pseudostratified epithelia, such as the mammalian neuroepithelium, are comparatively understudied. Pseudostratified epithelia also undergo oscillatory nuclear migration as cells progress through the cell cycle, known as interkinetic nuclear migration (INM). Nuclear movement during INM is believed to proceed in phases: active microtubule-dependent nuclear ascent towards the apical surface during G2 followed by actin-dependent cell rounding in M phase and ‘passive’ nuclear descent towards the basal surface during G1/S (Kosodo et al., 2011; Leung et al., 2011; Spear and Erickson, 2012). Progression of INM also influences the dimensions of the apical portion of a cell. During S phase, nuclei are basally located and the apical surface is small, mimicking apically constricted wedge-shaped cells, whereas nuclei are larger and apically located during mitosis, presumably producing larger apical surfaces (Guthrie et al., 1991; Nagele and Lee, 1979).

Both INM and apical constriction occur in the pseudostratified neuroepithelium of the closing neural tube. Failure of neural tube closure causes severe congenital defects, such as spina bifida, in ∼1:1000 births (Cavadino et al., 2016). Spina bifida arises due to failure of the open caudal segment of the neural tube, the posterior neuropore (PNP), to undergo the narrowing and shortening required for closure. PNP closure is fundamentally a biomechanical event during which the flat neural plate elevates lateral neural folds that buckle at paired dorsolateral hinge points. The neural folds become apposed medially, such that their tips meet at the dorsal midline where they are then joined by cellular protrusions that ‘zipper'down the length of the neuropore (Nikolopoulou et al., 2017). PNP narrowing through neural fold medial apposition involves both apical constriction and INM. Regional prolongation of S phase in the neuroepithelium along the PNP midline results in the accumulation of wedge-shaped cells, bending the tissue at the medial hinge point (McShane et al., 2015; Smith and Schoenwolf, 1988). Unlike pulsatile apical constrictions, this hinge point is stable and persists at the tissue level throughout most of PNP closure (Shum and Copp, 1996).

PNP closure can be expected to fail if its tissue structures are abnormal, if pro-closure cell-generated mechanical forces cannot exceed forces which oppose closure or if those forces are not transmitted in a coordinated manner across the PNP. We have recently reported two genetic mouse models in which excessive tissue tensions opposing PNP closure predict failure of closure and development of spina bifida (Galea et al., 2017, 2018). Tissue tension was inferred from physical incision or laser ablation experiments in which the most recently fused portion of the neural tube, the zippering point, was disrupted and the resulting rapid deformation of the PNP quantified (Galea et al., 2017, 2018). These experiments also showed that the PNP is a biomechanically coupled structure thanks at least in part to supracellular actomyosin cables that run rostro-caudally along the tips of the neural fold (Galea et al., 2017, 2018). Hence, ablation of the PNP zippering point causes neuropore widening, which extends into more posterior portions of the open region. The apical neuroepithelium also forms distinct supracellular F-actin enrichments (‘profiles’) that are oriented mediolaterally, in the direction of neural fold apposition (Galea et al., 2018; Nishimura et al., 2012). Consistent with the involvement of specialised F-actin structures in PNP closure, inhibition of the actomyosin regulator Rock with the commonly used antagonist Y27632 stalls PNP closure in mice and other vertebrates (Escuin et al., 2015; Kinoshita et al., 2008).

Rock inhibition impairs the selective apical enrichment of actomyosin required for apical constriction in the neuroepithelium (Escuin et al., 2015) and other tissues (Harding and Nechiporuk, 2012; Sai et al., 2014). We set out to test whether stalling of PNP closure in Rock-inhibited embryos is caused by lack of apical constriction, or whether it involves failure of alternative force-generating mechanisms, such as INM. In testing this hypothesis, we investigated a more fundamental question: how are apical constriction and INM functionally coordinated to regulate apical area in neuroepithelial cells?

## RESULTS

### Rock inhibition widens the PNP and diminishes the neural fold actomyosin cables

**Fig. 1.**
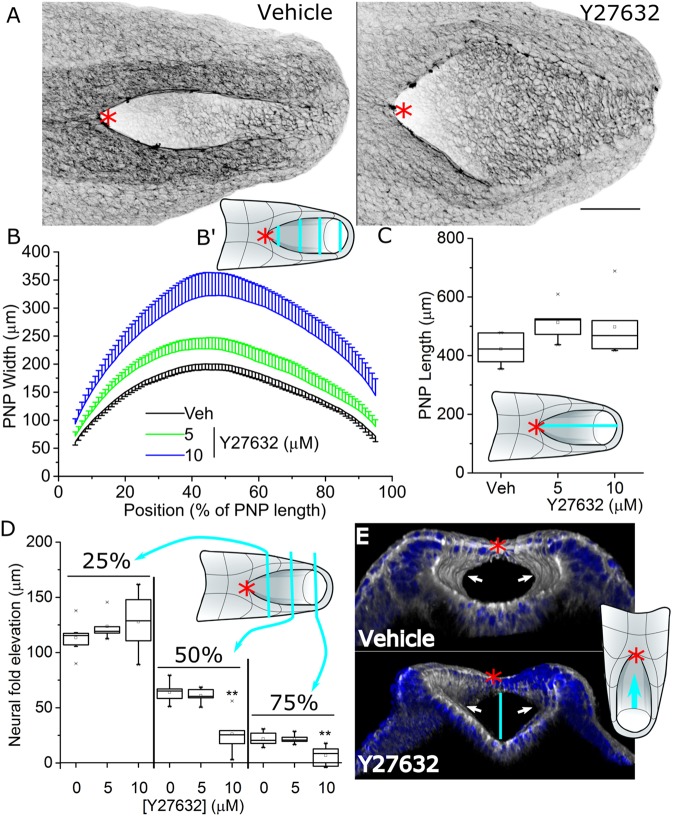
**Rock inhibition widens the PNP and reduces** **neural fold elevation.** E9 CD1 embryos were cultured in vehicle (Veh, *n*=6) or with the indicated concentrations of Y27632 (5 µM, *n*=5; 10 µM, *n*=6) for 8 h. (A) Representative wholemount phalloidin-stained vehicle and 10 µM Y27632-treated embryo PNPs (dorsal view). Scale bar: 100 µm. Image is presented after applying an inverted greyscale look-up table. (B) Sequential quantification of PNP width at every 1% of its length. The schematic in B′ illustrates sequential width measurements shown by the cyan lines across the PNP. (C) Quantification of PNP length as shown by the cyan line in the schematic. (D) PNP elevation was quantified as the dorsoventral distance between the neural fold tips and apical surface of the midline neuroepithelium (vertical line in E) at 25%, 50% and 75% of the length of the PNP. (E) 3D-reconstructed images of a vehicle and 10 µM Y27632-treated embryo PNP, illustrating the presence of dorso-lateral hinge points in both. These reconstructions are shown looking rostrally into the closure neural tube as indicated by the cyan arrow in the schematic. The red asterisks denote the zippering point throughout. **P<0.01 (tests defined in Materials and Methods); embryos were analysed at the 19–22 somite stage.

Prolonged Rock inhibition at concentrations compatible with continued development in mouse whole embryo culture delays PNP closure, producing longer PNPs than in vehicle-treated embryos (Escuin et al., 2015). To minimise the potential for secondary changes owing to prolonged culture, we first characterised the morphological changes caused by 8 h of Rock inhibition with the extensively used compound Y27632 in embryonic day (E)9–9.5 CD1 mouse embryos. This treatment period is sufficient to observe biologically meaningful differences in PNP dimensions (Hughes et al., 2018). After 8 h of Rock inhibition, we observed dose-dependent widening of the PNP, giving rise to PNPs that were more ‘diamond-shaped’ as opposed to the the elliptical structures characteristic of control embryos at late stages of closure (Fig. 1A,B). This short period of Rock inhibition did not significantly increase PNP length (Fig. 1C). Neural fold elevation tended to be more variable in Rock-inhibited than vehicle-treated embryos (Leven's test *P*=0.10), but was not significantly altered by Rock inhibition in the region near the zippering point (25% of the length of the PNP from the rostral end, Fig. 1D). Neural fold elevation was significantly reduced caudal to this location (Fig. 1D). Dorsolateral hinge points were still present in Rock-inhibited embryos (Fig. 1E), as previously reported (Escuin et al., 2015). These morphometric studies reveal tissue shape changes caused by 8 h of Rock inhibition, of which PNP widening is the most marked, for which biomechanical mechanisms were further investigated.

**Fig. 2.**
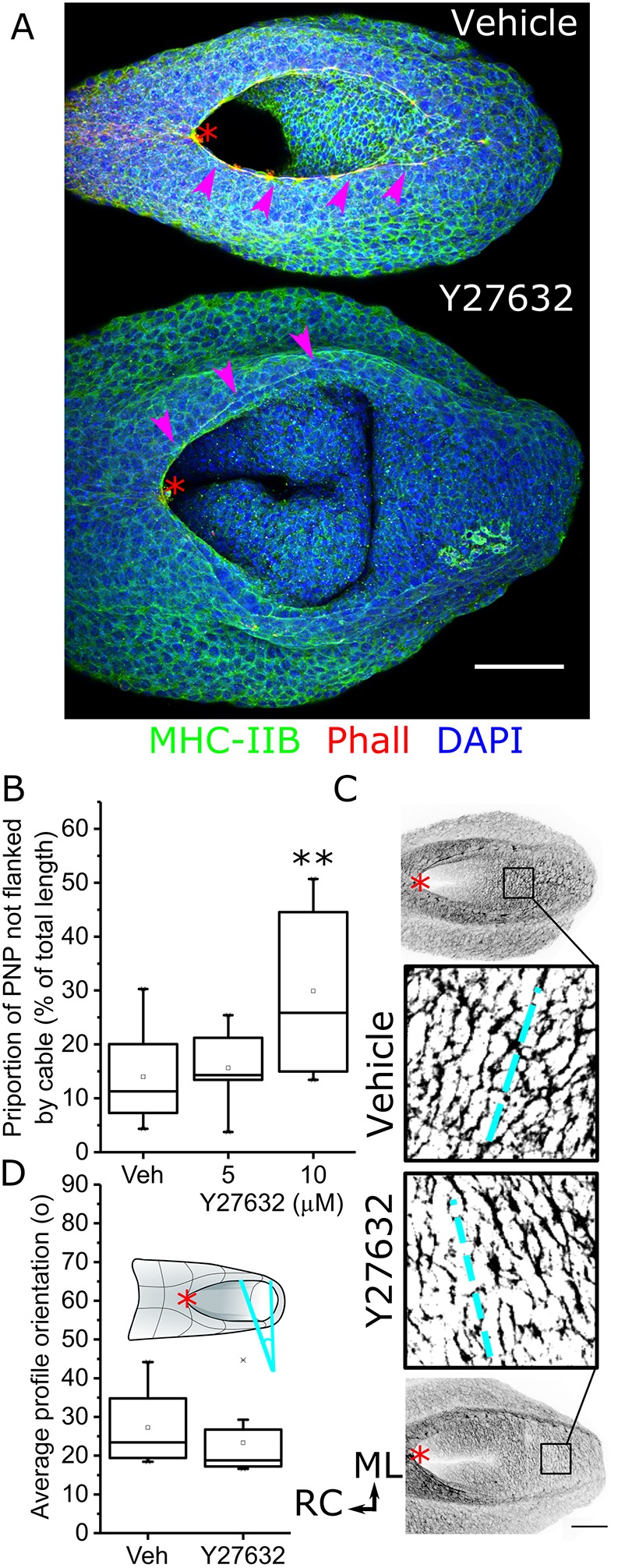
**Rock inhibition diminishes the rostrocaudal neural fold F-actin cables but not mediolateral neuroepithelial profiles.** E9 CD1 embryos were cultured in vehicle or the indicated concentrations of Y27632 for 8 h. (A) Representative wholemount-stained vehicle- and 10 µM Y27632-treated embryo PNPs (dorsal view). Arrowheads indicate the rostrocaudal actomyosin cables. (B) Quantification of the proportion of the PNP which extends beyond the caudal limit of the rostrocaudal cables, as previously defined (Galea et al., 2017; Hughes et al., 2018). Vehicle, *n*=8; 5 µM, *n*=5; 10 µM, *n*=7. (C) Visualisation of mediolateral F-actin profiles (image is presented after applying an inverted grey look-up table, which is binarised in the magnified views) in the relatively flat portion of the PNP in vehicle- and 10 µM Y27632-treated embryos. Dashed lines indicate the mean orientation quantified for those embryos. (D) Quantification of the mean orientation of binarised F-actin profiles in vehicle (*n*=8) and 10 µM Y27632-treated embryos (*n*=8) relative to the mediolateral direction (cyan angle bracket in the schematic). The red asterisks denote the zippering point throughout. Scale bars: 100 µm. **P<0.01 (ANOVA with post-hoc Bonferroni); embryos were analysed at the 19–22 somite stage.

As previously reported (Escuin et al., 2015), Rock inhibition diminished the selective localisation of F-actin in the apical neuroepithelium (Fig. S1). In addition, we specifically investigated two supracellular F-actin organisations present in the PNP (Galea et al., 2017, 2018): long rostrocaudal cables along the neural folds (Fig. 2A) and mediolateral profiles identifiable in the apical neuroepithelium (Fig. 2C). Although rostrocaudal F-actin cables remained evident close to the zippering point, the proportion of the PNP not flanked by these cables was significantly greater in embryos treated with 10 µM Y27632 for 8 h than those cultured in vehicle (Fig. 2A,B; also see Fig. 1A). Unexpectedly, mediolaterally oriented supracellular F-actin profiles were still evident in the neuroepithelium of the open PNP of Rock-inhibited embryos (Fig. 2C). The average orientation of F-actin profiles in each PNP was not significantly different between vehicle and Rock-inhibited embryos (Fig. 2C,D).

### Rock inhibition diminishes PNP tissue tension

**Fig. 3.**
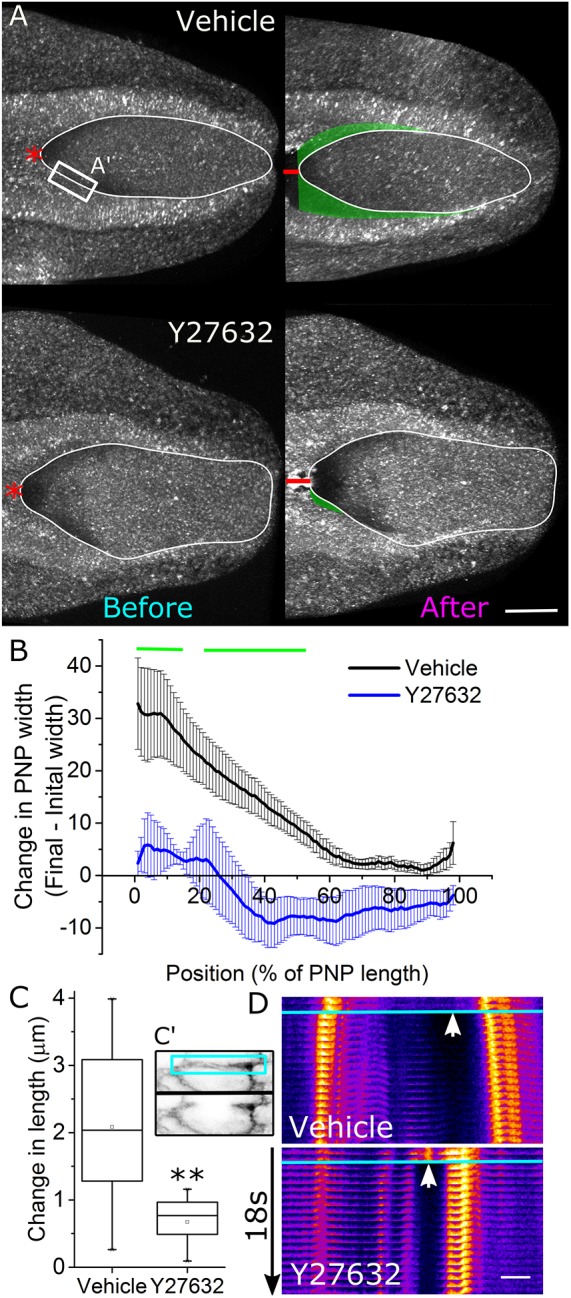
**Rock inhibition diminishes anti-closure PNP tissue tension.** (A) Representative reflection live-imaged PNPs following 8 h of culture in vehicle or 10 µM Y27632. Each neuropore was imaged before and again after laser ablation (red line) of the zippering point. The white perimeter indicates the shape of the PNP before ablation, the green-shaded region indicates lateral displacement of the neural folds. Ablations of the rostrocaudal F-actin cables were performed in different embryos in the region indicated by A′. Scale bar: 100 µm. The red asterisks denote the zippering point. (B) Mediolateral change in width of the PNP in vehicle- (*n*=7) and 10 µM Y27632-treated embryos (*n*=6) at each 1% of the length of the PNPs from the zippering point (position 0%). Green lines indicate the region in which vehicle-treated embryos recoiled to a significantly greater extent than 10 µM Y27632-treated embryos (*P*<0.05, mixed model testing). (C) Rostrocaudal change in length (example in C′) of cell borders along the neural folds following laser ablation. *n*=9 per group, **P<0.01 (*t*-test). (D) Representative kymographs (from cell borders equivalent to the cyan box in C′) of cable ablations in vehicle- and 10 µM Y27632-treated embryos. Bright spots in each temporal slice are cell membranes on either side (left and right) of the ablation. White arrows indicate the ablated border, and temporal slices below the horizontal cyan line show displacement after laser ablation. Scale bar: 5 µm, kymographs were generated in Fiji.

Diminished rostrocaudal F-actin cables are associated with increased tissue tension in *Zic2^Ku/Ku^* embryos, as shown by greater tissue recoil following zippering point ablation (Galea et al., 2017). In contrast, Rock inhibition for 8 h prior to and during zippering point ablation substantially diminished lateral tissue recoil (Fig. 3A,B). The caudal-open PNP of Rock-inhibited embryos appeared to narrow following zippering point ablation, although this did not reach significance at any position. These findings suggest Rock inhibition decreases tissue tensions that pull the neural folds laterally.

We next analysed cell-level tension by quantifying recoil from targeted laser cuts of the cell borders on the neural folds along which the rostrocaudal cables normally run. These ablations were performed close to the zippering point, where F-actin cables could typically still be identified in Rock-inhibited embryos. Ablated borders in vehicle-treated embryos elongated by ∼2 µm immediately following ablation (Fig. 3C). Border recoil following laser ablation in Rock-inhibited embryos was less than half of that quantified in vehicle-treated controls (Fig. 3C,D), corroborating reduced tension following Rock inhibition. Collectively, these tissue- and cell-level laser ablation experiments suggest that PNP widening in Rock-inhibited embryos is not caused by increases in tissue tensions pulling on the zippering point.

**Fig. 4.**
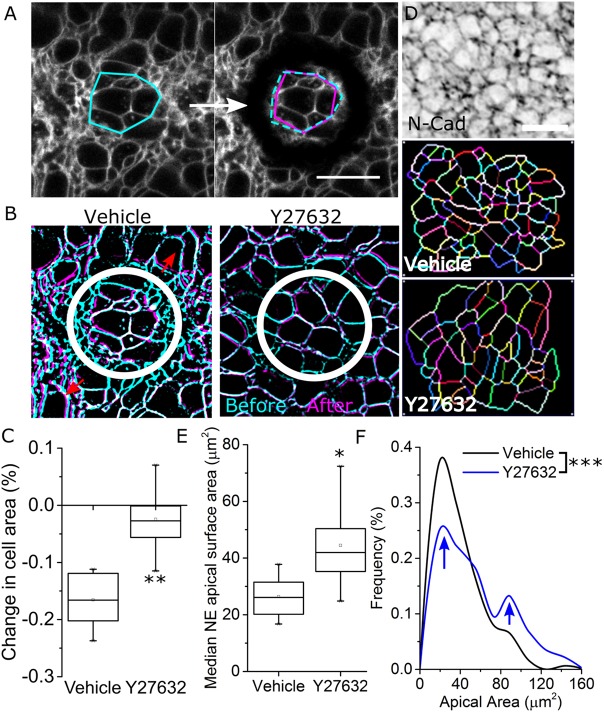
**Rock inhibition reduces neuroepithelial apical constriction.** (A) Representative annular ablation in the region indicated by the white circle in the representative whole-PNP view, imaged before and immediately after ablation. A single ablation was performed in each embryo. The area within the ablated circle is shown by the cyan polygon between definable landmarks before ablation, which deformed to the magenta polygon immediately following ablation. Scale bar: 25 µm. (B) Representative segmented and registered cell borders before (cyan) and immediately after (magenta) annular ablation (white circle) illustrating their displacement in both vehicle- and 10 µM Y27632-treated embryos. The red arrows illustrate that the surrounding tissue also retracts away from the ablation. (C) Quantification of the constriction of the tissue within the ablated circle in vehicle- (*n*=8) and 10 µM Y27632-treated (*n*=9) embryo PNPs. (D) Surface-subtracted N-cadherin staining from a vehicle-treated embryo. Scale bar: 20 µm. Cell borders were segmented using Tissue Analyser as illustrated. (E) Quantification of median apical areas of neuroepithelial cells based on segmented N-cadherin staining in vehicle- and 10 µM Y27632-treated embryos (*n*=6 each) following 8 h of culture. (F) Frequency plot of observed apical areas of neuroepithelial cells in vehicle- (324 cells from 6 embryos) and 10 µM Y27632-treated embryos (244 cells from six embryos). The arrows indicate that although the majority of cells retain small apical areas despite Rock inhibition, there is a highly significant shift towards more cells having large apical areas. *P<0.05, **P<0.01, **P<0.001 (tests defined in Materials and Methods).

We next sought to determine whether Rock inhibition alters tension within the neuroepithelium of the open PNP. This posed a challenge as, unlike simpler epithelia, neuroepithelial cells do not have predictable straight borders that can be reproducibly visualised and ablated (Fig. S2A′). We initially developed a method of performing long, linear laser ablations along the apical neuroepithelium and quantified lateral retraction (Fig. S2A,B). While this confirmed the neuroepithelium is under tension, recoil magnitudes varied substantially along the length of the ablation (Fig. S2B). A pilot study of five comparable embryos ablated on the same day showed this method was too variable to allow meaningful comparisons between treatment groups (sample size calculation based on pilot study quantifying mid-ablation recoil in five embryos required 90 embryos to detect a 30% difference with 80% power at *P*<0.05). To circumvent these issues, we developed a novel method in which a 30-µm-diameter annular ablation is created in the neuroepithelium, isolating a cluster of approximately eight cells (Fig. 4A). The reduction in area of this cluster of cells as they constrict immediately following ablation was then quantified as a readout of tension (Fig. 4A–C, sample size calculation based on pilot study of five embryos required approximately eight embryos to detect a 30% difference with 80% power at *P*<0.05). Cell constriction following annular ablations was significantly greater in vehicle-treated than Rock-inhibited embryos, suggesting that Rock inhibition reduces tension in the neuroepithelium (Fig. 4C).

To establish whether this reduction in neuroepithelial tension correlates with diminished apical constriction, apical areas were quantified in N-cadherin-stained PNPs (Fig. 4D). The median apical area for the neuroepithelial cells were significantly larger in Rock-inhibited than in vehicle-treated embryos (Fig. 4E). However, the distribution of apical area observed in this epithelium was highly skewed. Frequency versus apical areas plots were used to analyse shifts in apical area across the cell population. Leftward shifts in the frequency curve indicate a greater proportion of cells in the population have small apical areas, whereas rightward shifts indicate a greater proportion of cells have large apical areas. In Rock-inhibited embryos the proportion of cells with large apical areas increased, producing a significant shift in observed dimensions towards larger sizes (short arrow in Fig. 4F), although the majority of cells retained small apical areas despite Rock inhibition (long arrow in Fig. 4F). Taken together, these data suggest that the increase in the neuroepithelial apical size in Rock-inhibited embryos is not caused by ‘stretching’ given tissue tension is diminished. The simultaneous increase in apical size and reduction in mechanical tension is consistent with Rock inhibition stopping apical constriction in at least a subpopulation of cells in the mammalian neuroepithelium.

### The apical area of neuroepithelial cells decrease during exit from M phase

**Fig. 5.**
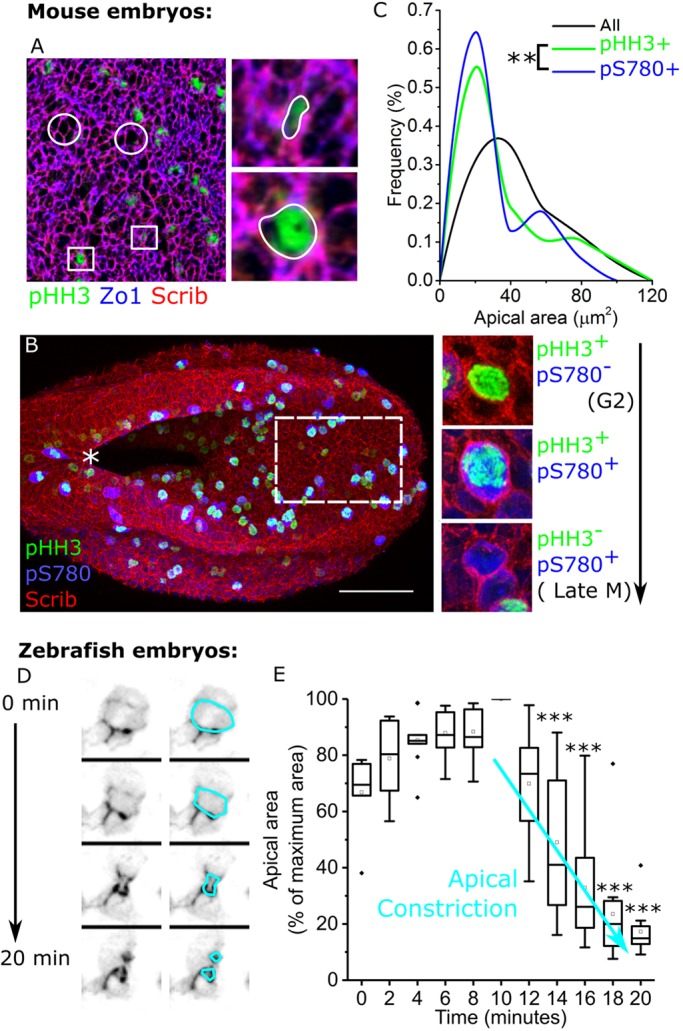
**Neuroepithelial cells undergo apical re-constriction in late M phase.** A–C represent data from non-cultured mouse PNPs, whereas D and E are from live-imaged zebrafish hindbrain neuroepithelium. (A) Representative triple-labelled neuroepithelial apical surface showing ZO-1 (apical-most tight junction marker), Scrib and the G2/M phase marker pHH3. Circles indicate cells with large apical areas that are negative for pHH3. Squares indicate pHH3^+^ cells with large and small apical areas, as shown in the magnified views. (B) Representative wholemount maximum projection showing the pattern of Scrib, pHH3 and pS780 staining in an uncultured embryo. The dashed white box indicates the relatively flat region of the PNP caudal to the medial hinge point in which apical areas were analysed. Optical cross-sections through pHH3/pS780 single and double positive cells (which appear cyan) are also shown. The white asterisk denotes the zippering point. Scale bar: 100 μm. (C) Frequency plot showing the distribution of apical area (based on Scrib staining) in the overall neuroepithelial cell population (‘All’, 262 cells from 5 embryos), and for pS780-positive (69 cells) and pHH3-positive cells (58 cells). **P<0.01 (Kolmogorov–Smirnov test). (D) Representative snapshots of a live-imaged zebrafish hindbrain neuroepithelium, with the apical cell surface mosaically labelled with Par3–RFP (cyan outline), undergoing apical reconstriction prior to division. The last time point shown (post-division) was not included in the apical area analyses shown in E. (E) Quantification of apical area for cells in the zebrafish hindbrain neuroepithelial over time. For each cell, its maximum size prior to division was identified (set at 100% for each cell) and 10 time points were analysed around this maximum dimension (i.e. *t*=10 min set at 100%). *n*=14 divisions from seven embryos. ***P<0.001 versus the maximum dimension (repeated measures ANOVA with Bonferroni post-hoc).

The presence of larger apical surfaces in neuroepithelial cells, which take up more space, may explain why Rock inhibition widens the PNP. Given that apical surfaces widen as nuclei approach the apical side during INM, we hypothesised this may be related to accumulation of mitotic cells with large apical surfaces in Rock-inhibited embryos. Rock1 protein is normally enriched around the apices of neuroepithelial cells, including mitotic cells positive for phosphorylated histone 3 (pHH3) (Fig. S3A), but Rock inhibition did not substantially alter the neuroepithelial mitotic index (Fig. S3B). Furthermore, neuroepithelial cells with the largest observed apical areas were not necessarily positive for the G2/M phase marker pHH3 (Fig. 5A). This was visualised in uncultured embryos triple-labelled in whole-mount for pHH3, ZO-1 (also known as TJP1) to show the apical surface, and scribble (Scrib) to label basolateral cell borders so that individual nuclei could be definitively related to their own apical surface. Scrib recapitulated ZO-1 staining at the apical surface (Fig. 5A), so Scrib staining was used to analyse apical area in subsequent experiments. These analyses showed that although pHH3 sometimes labels cells with a large apical area, it can also label cells with small apices (Fig. 5A). 3D cell reconstructions showed that pHH3^+^ cells can have small apical areas relative to their pHH3-negative neighbours. Non-mitotic neighbours appear to wrap over pHH3^+^ cell bodies (Movie 1), maintaining continuity of apical junctions.

The commonly used S10-phosphorylated pHH3 is not a pan-M phase marker; cells are positive for this marker from G2 to anaphase (Dai et al., 2005; Ren et al., 2018). To extend our analysis we stained for the pan-M phase phosphorylated S780 epitope of retinoblastoma protein (an Aurora B kinase target site; Macdonald and Dick, 2012; Nair et al., 2009), which produces bright staining (hereafter denoted pS780^+^) throughout M phase, persisting beyond anaphase to the end of cytokinesis (Jacobberger et al., 2008) (see Fig. 5B). In order to more closely analyse the distribution of apical areas around the time of mitosis, we triple-labelled PNPs for Scrib, pHH3 and pS780.

The apical areas of pHH3^+^ (G2 to anaphase) or pS780^+^ (M phase to cytokinesis) neuroepithelial cells were analysed in the relatively flat region of the PNP caudal to the median hinge point of non-cultured mouse embryos. Single versus double positivity for these markers could not be taken into account given the small number of cells labelled with either, typically ∼15 per PNP. pHH3^+^ cells included those with the largest apical area observed, but the majority had small apical surfaces (Fig. 5C, mean apical area for ‘All’ cells, 34.3 µm^2^; pHH3^+^, 26.0 µm^2^). The distribution of pS780^+^ apical surfaces was significantly shifted towards smaller dimensions relative to pHH3^+^ cells (Fig. 5C, mean of 20.4 µm^2^). A small proportion of pS780^+^ cells will have recently completed division.

These findings suggest that apical areas of neuroepithelial cells typically decrease as cells transition through M phase towards G1. In order to dynamically visualise this apical re-constriction at the end of mitosis, we live-imaged the hindbrain neuroepithelium of zebrafish embryos (Fig. 5D). Mitotic cells were identified as those which completed division to form two daughter cells while being imaged and their apical areas over ∼20 min prior to division were analysed (Fig. 5D). In this species, we found that apical sizes in neuroepithelial cell increased to a maximum size in early mitosis, then rapidly re-constricted prior to division (Fig. 5E). Thus, end-mitotic apical re-constriction is an evolutionarily conserved neuroepithelial cell behaviour in fish and mice.

### Rock inhibition preferentially increases apical area in late M phase cells

**Fig. 6.**
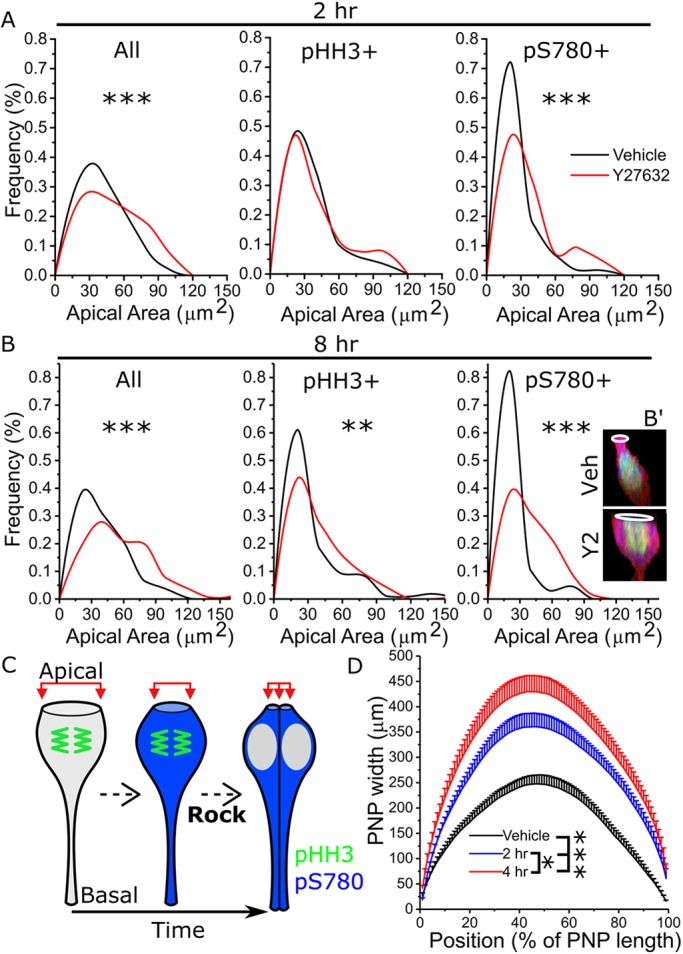
**Increases in apical area of neuroepithelial cells following Rock inhibition are cell cycle stage specific.** (A,B) Embryos were cultured for (A) 2 h or (B) 8 h in vehicle or 10 µM Y27632 and triple-stained for Scrib, pHH3 and pS780. Apical areas were analysed in the overall cell population (‘All’), in all pHH3^+^ cells and in all pS780^+^ cells. B′ illustrates the shape (red, Scrib-labelled cell borders) and apical area (white rings) of a pHH3/pS780 double-positive cell from a vehicle- and Y27632-treated embryo. *n* numbers are as follows: All, 2 h vehicle, *n*=310 cells from six embryos, Y27632, *n*=267 cells from six embryos; All, 8 h vehicle, *n*=253 cells from five embryos, Y27632, *n*=320 cells from seven embryos; pHH3^+^, 2 h vehicle, *n*=87 cells, Y27632, *n*=80 cells; pHH3^+^, 8 h vehicle, *n*=110 cells, Y27632, *n*=102 cells; pS780^+^, 2 h vehicle, *n*=118 cells, Y27632, *n*=97 cells; pS780, 8 h vehicle, *n*=68 cells, Y27632, *n*=84 cells. (C) Schematic of the proposed model of apical constriction of neuroepithelial cells as cells transition from early M (pHH3^+^/pS780^−^) through cytokinesis into G1 (pHH3^−^/pS780^+^). Rock-dependent constriction is indicated in late M phase. (D) Sequential quantification of PNP width at every 1% of its length in embryos cultured in vehicle (2 h culture) or 10 µM Y27632 for 2 h or 4 h (*n*=6 embryos per group analysed at the 16–20 somites stage). *P<0.05, **P<0.01, ***P<0.001 (tests defined in Materials and Methods).

To determine whether Rock-dependent apical constriction is specific to a cell cycle phase, we initially analysed the apical area of pHH3^+^ or pS780^+^ cells after 2 h of Rock inhibition. This duration of treatment was selected because it is sufficient for significant PNP narrowing to occur (Galea et al., 2017), but not for cells to progress through a complete cell cycle (McShane et al., 2015). This Rock inhibition was sufficient to increase the proportion of cells with large apical areas in the overall population (Fig. 6A, All). Remarkably, the apical areas of pHH3^+^ cells were not significantly altered by Rock inhibition (Fig. 6A, pHH3^+^), whereas apical areas of pS780^+^ cells were significantly larger in Rock-inhibited than vehicle-treated embryos (Fig. 6A, pS780^+^). Consequently, following Rock inhibition the observed apical area frequencies of pS780^+^ cells approximated those of pHH3^+^ cells (Fig. 6A, pHH3^+^ versus pS780^+^).

Following 8 h of treatment, Rock inhibition significantly increased apical areas overall as well as in both the pHH3^+^ and pS780^+^ populations (Fig. 6B). Rock inhibition did not significantly change the proportion of cells labelled with pHH3 or pS780 individually or together (Fig. S3C), further suggesting that Rock inhibition minimally affects mitotic progression in this epithelium. Blocking the Rock-dependent reduction in apical area as cells transition from G2 to G1 may therefore have cumulative effects as cells continue to progress through the cell cycle (Fig. 6C), progressively widening the PNP. Consistent with this, whereas 2 h of Rock inhibition was sufficient to significantly widen the PNP (or prevent its narrowing in culture), PNP width increased further following an additional 2 h of inhibition (4 h total, Fig. 6D).

### Blocking cell cycle progression prevents PNP widening caused by Rock inhibition

**Fig. 7.**
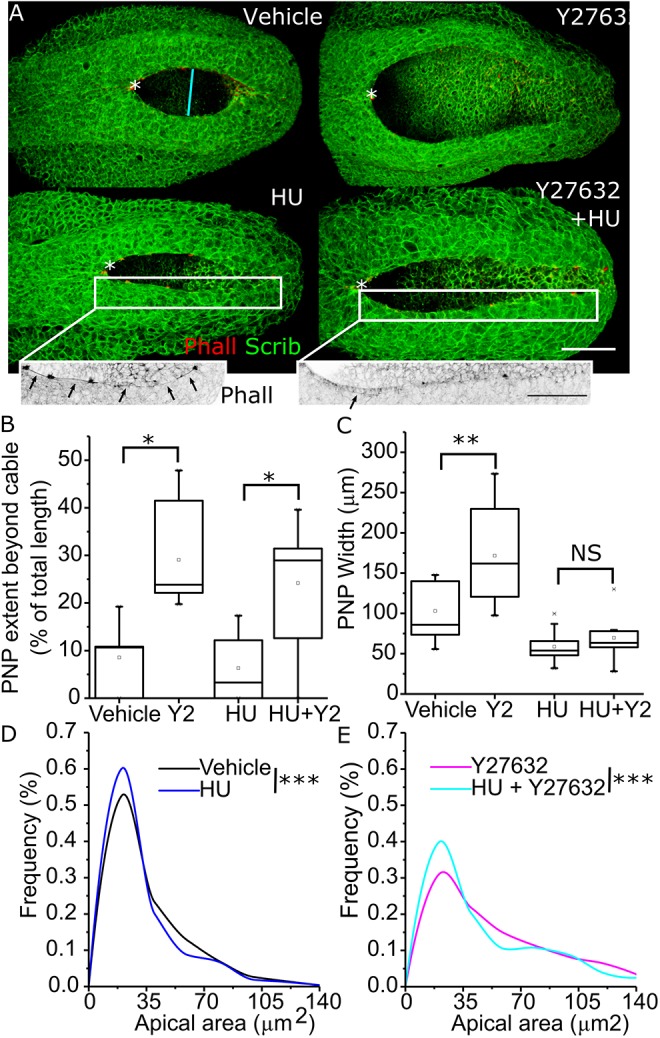
**Cell cycle progression is a prerequisite for PNP widening in Rock-inhibited embryos.** (A) Representative 3D-rendered PNP images from embryos treated with vehicle, 10 µM Y27632 (Y2), 0.8 mM HU or HU+Y27632 after 8 h of culture. The asterisks indicate the zippering point, cyan lines approximate the mid-PNP width. The insets below the HU and HU+Y27632-treated embryos are presented after applying an inverted grey look-up table for phalloidin staining to facilitate visualisation of the rostrocaudal F-actin cables (arrows). Scale bars: 100 µm. (B) Quantification of the proportion of the PNP which extends beyond the rostrocaudal cables (as in Fig. 2B) in each treatment group. (C) Quantification of mid-PNP width in each treatment group. Embryos were analysed at the 20–23 somites stages; vehicle, *n*=6; Y27632, *n*=7; HU, *n*=9; HU+Y27632, *n*=9. (D,E) Apical areas of neuroepithelial cells were analysed in ZO-1-stained PNPs from (D) vehicle versus HU-treated and (E) 10 µM Y27632- versus HU+Y27632-treated embryos after 8 h of culture. *n* numbers were: vehicle, *n*=845 cells from six embryos, HU, *n*=816 cells from seven embryos; Y2, *n*=861 cells from six embryos, HU+Y2, *n*=901 cells from six embryos. NS, not significant; *P<0.05, **P<0.01, ***P<0.001 (tests defined in Materials and Methods).

The findings that Rock inhibition increases neuroepithelial apical areas at specific cell cycle phases in a temporally restricted manner suggest that progression through the cell cycle contributes to PNP widening in Rock-inhibited embryos. This predicts that inhibition of progression through the cell cycle may diminish PNP widening following Rock inhibition. To test this, we treated embryos with the ribonucleotide reductase inhibitor hydroxyurea (HU) which blocks cell entry into S phase (Leitch et al., 2016; Philips et al., 1968). As well as being used therapeutically for various conditions in humans, HU has previously been reported to reduce spina bifida incidence in the *curly tail* mouse model *in vivo* (Seller and Perkins, 1983). In the present study, treatment with 0.8 mM HU for 8 h substantially, but not completely, diminished the neuroepithelial mitotic index (Fig. S4A,B). Reducing cell cycle progression with HU did not restore the rostrocaudal F-actin cables (Fig. 7B), but fully prevented PNP widening after 8 h of Rock inhibition (Fig. 7A,C). This is consistent with a requirement for cell cycle progression during PNP widening due to Rock inhibition.

Diminished cell cycle progression in HU-treated embryos precluded analysis of cell cycle phase-specific apical areas. Given that HU treatment blocks cells in S phase (Leitch et al., 2016; Philips et al., 1968), when they have small apical areas (Guthrie et al., 1991; Nagele and Lee, 1979), a greater proportion of neuroepithelial cells had small apical areas after 8 h of HU treatment (Fig. 7D), as expected. Rock inhibition increased apical areas in a subset of neuroepithelial cells independantly of cell cycle progression, but HU treatment partly prevented this increase (Fig. 7E). Thus, blocking cell cycle progression produces fewer cells (fewer divisions) with smaller apical sizes than Rock inhibition alone, preventing progressive PNP widening.

## DISCUSSION

Tissue-level integration of cellular force-generating mechanisms is necessary to achieve coordinated morphogenetic shape change. Force-generating mechanisms often act in opposing ways; for example, both cell apoptosis (Oltean and Taber, 2018) and regional cell proliferation (Hosseini et al., 2017; Peeters et al., 1998) can change tissue shape despite having opposing effects on cell number. This mutual antagonism may also be true of the interplay between apical constriction and INM. Here, we assessed the functional integration of these force-generating mechanisms in the mammalian neuroepithelium by using a highly reproducible model of PNP closure suppression through pharmacological inhibition of Rock. Pharmacological antagonism allows greater temporal control over Rock activity than can currently be achieved genetically in mouse embryos. Small molecule Rock inhibitors are in clinical development for conditions ranging from glaucoma to cardiovascular disease (Hartmann et al., 2015; Honjo and Tanihara, 2018). In the present study, we demonstrate that Rock is required for supracellular organisation of F-actin into biomechanically coupling rostrocaudal cables, and document marked progressive PNP widening following Rock inhibition.

Rock inhibition diminishes F-actin radial and stress fibres in immature epidermis-derived epithelia, but has minimal effects on F-actin organisation in epithelia matured *in vitro*, suggesting Rock-independent F-actin organisation in epithelial cells with mature intercellular junctions (Vaezi et al., 2002). In the present study, mediolaterally oriented F-actin profiles remained evident in the neuroepithelium of Rock-inhibited mouse embryos. This is consistent with the recent finding that directional supracellular F-actin enrichments in stretched *Drosophila* wing disks also form independently of Rock (Duda et al., 2019). PNP mediolateral profiles require planar cell polarity (PCP) signalling (Galea et al., 2018; McGreevy et al., 2015) and, while Rock has been suggested to mediate downstream events of PCP signalling in some contexts (Winter et al., 2001), our findings suggest that this PCP-regulated event is relatively independent of Rock signalling. Conditional mosaic deletion of the mammalian core PCP component Vangl2 (Galea et al., 2018), or compound heterozygous mutations of Vangl2 and the Diaphanous-related formin Daam1, lead to spina bifida (Lopez-Escobar et al., 2018). Diaphanous 1 is required for Rock-independent polarised F-actin profiles to form in stretched *Drosophila* wing disks (Duda et al., 2019), suggesting a Rock-independent PCP pathway directs mechanoresponsive actomyosin organisation.

PCP mutations prevent convergent extension movements, but cell migration is unlikely to substantially contribute to the rapid increase in PNP width in Rock-inhibited embryos. PNP widening could be caused by increases in laterally tethering mechanical tensions, as inferred from rapid lateral recoil of the neural folds following laser ablation of the zippering point. Increased recoil precedes failure of PNP closure and development of spina bifida in *Zic2^Ku/Ku^* embryos and in embryos with a conditional knockout in *Vangl2* (Galea et al., 2017, 2018). The opposite is seen in Rock-inhibited embryos; recoil is substantially diminished compared with vehicle-treated controls. These experiments may be limited by differences in tissue material properties, potentially including a reduction in neuroepithelial material stiffness in Rock-inhibited embryos (Nagasaka et al., 2016). However, structural stiffness of another embryonic structure, the *Xenopus* blastopore, is insensitive to short-term Rock inhibition (Feroze et al., 2015). Structural differences in PNP morphology might also confound tissue-level analysis, although Rock inhibition minimally altered structural features, such as dorsolateral hinge points and neural fold elevation close to the zippering point. Our cell-level analyses also demonstrated reduced recoil following laser ablation of the cell borders along which the rostrocaudal actomyosin cables run. Taken together, these findings exclude an increase in tissue tensions as a biomechanical explanation for PNP widening in Rock-inhibited embryos.

As well as reducing laterally tethering tension, Rock inhibition also diminished apical constriction of neuroepithelial cells. Apical constriction of epithelial cells is commonly documented by visualising reductions in apical areas, as we and others have previously reported in the neuroepithelium (Bush et al., 1990; Galea et al., 2017; McGreevy et al., 2015). This cannot differentiate active force-generating apical constriction from external compression, or indeed cell shape changes linked to cell cycle progression in a pseudostratified epithelium. Here, we provide three levels of evidence showing that the cells from mammalian neuroepithelium undergoes apical constriction in a Rock-dependent manner: (1) apical F-actin localisation is lost, and (2) neuroepithelial apical areas are larger despite (3) diminished neuroepithelial tension in Rock-inhibited embryos. Previous work from our group has shown that inhibition of ATPase-dependent myosin-II activity with Blebbistatin rescues neuroepithelial F-actin apical localisation in Rock-inhibited embryos (Escuin et al., 2015). Blebbistatin itself did not impair PNP shortening, suggesting that sub-apical redistribution of F-actin potentially diminishes PNP closure by rendering the neural plate stiff and resistant to morphogenesis (Escuin et al., 2015). In the current study, we found that Rock inhibition globally and acutely diminishes PNP tension. This is consistent with the importance of actomyosin in establishing tissue tension prior to substantial extracellular matrix assembly in mammalian embryos, as previously reported in lower vertebrates (Porazinski et al., 2015). It remains unknown whether tissue tension in turn influences cell differentiation, as it does in other contexts (Martino et al., 2018).

Rock activity also regulates cell proliferation and cytokinesis in various contexts (Hazawa et al., 2018; Liang et al., 2019; Short et al., 2017), but no difference in mitotic indices were observed in Rock-inhibited neuroepithelial cells in the present study. Our findings confirm that mitotic neuroepithelial cells include those with the largest observed apical area in this epithelium. Unexpectedly, however, the mitotic population also includes neuroepithelial cells with some of the smallest apical areas, leading us to suggest that an apical re-constriction event happens during mitosis. Live-imaging of the zebrafish hindbrain confirmed this mitotic apical re-constriction occurs in neuroepithelial cells, and suggests it is conserved between two vertebrate species and anatomical sites.

Mitotic cell identification using pHH3 staining is limiting because this marker does not persist throughout M phase (Dai et al., 2005). Bright staining for the pS780 epitope used here overlaps with pHH3 in M (but not G2) phase and persists to the end of cytokinesis (Jacobberger et al., 2008). pS780^+^ cells have highly constricted apical areas, which occurs though a Rock-dependent mechanism, such that 2 h of Rock inhibition reverts the distribution of their apical areas to reflect that of pHH3^+^ cells. Remarkably, apical areas of pHH3^+^ neuroepithelial cells are insensitive to short-term Rock inhibition, suggesting that the Rock-dependent apical constriction process occurs at the end of M-phase. Rock-independent apical constriction has previously been described during *Drosophila* ventral furrow formation: pulsed treatment with Y27632 stopped the coordinated apical constriction required for furrow formation, but permitted the myosin II-independent slow reductions in apical area concomitant with apicobasal nuclear migration (Krajcovic and Minden, 2012). Possible Rock-independent mechanisms by which pHH3^+^ cells achieve small apical areas also include transfer of elastic energy (generated during G2 nuclear ascent) from neighbouring cells (Shinoda et al., 2018), consistent with the observed ‘wrapping’ of non-mitotic neighbours over pHH3^+^ cells. Alternatively, apical area may decrease in late M phase due to division of apical end feet into two daughter cells during cytokinesis. The absence of binucleated cells in this and previous (Escuin et al., 2015) studies suggests that Rock inhibition did not cause failure of cytokinesis in the neuroepithelium.

The apical area of pHH3^+^ cells did increase significantly following 8 h of Rock inhibition. Thus, in addition to the acute loss of apical constriction in late M phase, neuroepithelial cells with large apical areas accumulate as cells progress through the cell cycle while Rock activity is inhibited. The requirement for cell cycle progression to drive PNP widening in Rock-inhibited embryos is confirmed by the striking rescue achieved with HU treatment. Achieving rescue of PNP widening caused by Rock inhibition, which is both ubiquitous and pleiotropic, demonstrates the potential for a unified biomechanical understanding of morphogenesis to identify preventative interventions for structural malformations including neural tube defects. Taken together, our findings suggest a model of PNP neural fold apposition in which INM normally tends to widen the PNP due to the presence of large apical nuclei in G2 and early M phase. INM is counteracted by Rock-dependent apical constrictions as cells exit M phase, maintaining apical neuroepithelial tension.

## MATERIALS AND METHODS

### Embryo culture and treatments

Studies were performed under the regulation of the UK Animals (Scientific Procedures) Act 1986 and the Medical Research Council's Responsibility in the Use of Animals for Medical Research (1993). Outbred CD1 mice were bred in-house. Mice were mated during the day, and noon of the day a plug was found was considered E0. Pregnant females were killed in the morning of E9 (∼16 somites at the start of culture) and their embryos were cultured for 2–8 h. Embryo culture was performed using the roller bottle culture system in neat rat serum essentially as previously described by our group (Copp et al., 2000; Hughes et al., 2018). Embryos from each litter were approximately size-matched into groups, which were then randomly allocated to treatment groups using coin flips. Pharmacological agents were thoroughly mixed in culture rat serum prior to adding embryos. At the end of culture, embryos were dissected out of their extraembryonic membranes in the rat serum they were culture in, rinsed in ice-cold PBS and fixed in 4% PFA.

Zebrafish wild-type (AB/Tübingen) embryos were raised at 28.5°C in fish water or E2 medium containing 0.003% 1-phenyl-3-(2-thiazolyl)-2-thiourea (Sigma).

Y27632 was purchased from Cambridge Biosciences (SM02-1) and hydroxyurea was purchased from Sigma-Aldrich (H8627-1G). Both were dissolved in Milli-Q (MQ) water (vehicle). The concentrations and duration of treatment with each compound is stated in the results or figure legends.

### Wholemount staining, confocal microscopy and image analysis

Embryo wholemount staining and imaging were as previously described (Galea et al., 2017). Alexa-Fluor-568-conjugated Phalloidin was from Thermo Fisher Scientific (A12380), rabbit anti-MHC-IIb was from BioLegend (909901), goat anti-Scrib was from Santa Cruz Biosciences (SC-11049), rabbit anti-pS780-pRB (ab47763) and rabbit anti-Rock1 (ab45171) were from Abcam, rabbit anti-ZO-1 was from Thermo Fisher Scientific (402200), mouse anti-pS10-HH3 (‘pHH3’, 9706S), and mouse anti-N-cadherin (14215S) were from Cell Signalling Technology, all as previously validated by the manufacturers. All primary antibodies were used at 1:100–1:200 dilution. For N-cadherin and Rock1 staining, antigen retrieval was first performed by heating for 60 min on a 100°C hot plate in 10 mM sodium citrate with 0.05% Tween 20, pH 6.0. Alexa Fluor-conjugated secondary antibodies were from Thermo Fisher Scientific. Images were captured on a Zeiss Examiner LSM880 confocal using a 20×/NA 1.0 Plan Apochromat dipping objective. Whole PNP images were typically captured with *x*/*y* pixel sizes of 0.59 µm and a *z*-step of 1.0 µm (speed, 8; bidirectional imaging, 1024×1024 pixels). Images to analyse apical areas were captured with *x*/*y* pixel sizes of 0.21 µm and a *z*-step of 0.68 µm. Images were processed with Zen2.3 software and visualised as maximum projections in Fiji or as 3D reconstructions in Icy (whole PNPs) or Mesh Lab (individual cells) software.

To quantify the apical area of neuroepithelial cells in N-cadherin-stained PNP wholemounts, *z*-stacks were first surface subtracted to only show the apical 2–3 µm of tissue, and cell borders were segmented using Tissue Analyser (Aigouy et al., 2016) as previously described (Galea et al., 2018; macro available at https://www.ucl.ac.uk/child-health/core-scientific-facilities-centres/confocal-microscopy/publications). N-cadherin staining does not clearly demarcate cell borders, which is necessary to relate the apical area to cells in specific cell cycle stages. To do this, Scrib was used and apical areas were analysed in full *z*-stacks by identifying and manually drawing around individual apical surfaces of cells positive for either pHH3 or pS780. The marked differences in PNP morphology observed in Rock-inhibited embryos negated blinding to treatment group.

Morphometric comparisons were made using standard length measuring tools in Fiji. To analyse mediolateral F-actin profile enrichment, phalloidin-stained neuroepithelial wholemount maximum projections were first segmented by performing local contrast enhancement (CLAHE: 127 blocksize, 256 histogram bins on 16-bit images, 3 maximum slope), and then were binarised and despeckled. Average profile orientation was then calculated using the fit ellipse function in Fiji.

To live-image neuroepithelial divisions, zebrafish embryos were injected with Par3–GFP and Par3–RFP mRNAs as explained in Alexandre et al. (2010). Embryos at 24–30 h post fertilisation (hpf) were anaesthetised in MS-222 (Sigma), immobilised in 1% low-melting-point agarose and imaged using a LSM 880 (Zeiss) laser scanning confocal microscope and 20×/NA 0.95 water immersion objective. A series of small *z*-stacks (planes between 0.2–1 μm apart) were obtained every 1 to 2.5 min for 1–2 h. Data sets were prepared using Huygens deconvolution software and neuroepithelial apical areas were analysed by using Fiji software.

### Laser ablation

Zippering point laser ablations were performed as previously described using a MaiTai laser (SpectraPhysics Mai Tai eHP DeepSee multiphoton laser, 800 nm wavelength, 100% laser power, 65.94 µs pixel dwell time, 1 iteration). Reflection images of live embryo PNPs were obtained using a 10×/NA 0.5 Plan Apochromat dipping objective (633 nm laser wavelength). PNPs were imaged before and immediately after ablation, taking ∼3 min to capture each *z*-stack.

Whereas tissue-level zippering point ablations are intended to compromise a relatively large region of tissue quickly (∼250 µm long, ∼30 µm deep), cable and annular ablations were optimised to ensure targeted ablation of cell borders without vaporisation. Cable ablations were performed along a straight line of 0.1 µm wide at 710 nm wavelength, 80% laser power and 0.34 µs pixel dwell time for 20 iterations. Annular ablations were performed along a 30 µm diameter ring at 710 nm wavelength, 80% laser power and 0.34 µs pixel dwell time for 10 iterations. Vehicle- and Y27632-treated embryos in each experiment were alternately ablated in each experiment. Embryos were positioned in wells cut into agarose submerged in DMEM with 10% FBS immediately prior to ablation. Treated embryos were kept in 10 µM Y27632 throughout, including during the ablation.

### Statistical analysis

Comparisons between two groups were by undertaken with a Student's unpaired *t*-test accounting for homogeneity of variance in Excel or in SPSS (IBM Statistics 22). Comparison of multiple groups was undertaken with a one-way ANOVA or Kruskal–Wallis with post-hoc Bonferroni in OriginPro 2016 (Origin Labs). Multivariate analysis for serial PNP width or change in width measurements (following zippering point ablation) were undertaken with the linear mixed models in SPSS, accounting for the fixed effects of treatment and percentage of PNP length in repeated measures from each, with a post-hoc Bonferroni, as previously described (Galea et al., 2017). Frequency distributions are plotted in 20 µm bins and were compared using Kolmogorov–Smirnov tests. All images are representative of embryos from at least three independent experiments (defined as different litters processed on different days). Graphs were made in OriginPro 2016 (Origin Labs) and are represented as box plots, or as the mean±s.e.m. when several groups are shown per measurement level. For box plots, the box represents the 25–75th percentiles, and the median is indicated by a line and the mean by a square symbol. The whiskers show the 95% confidence intervals, and outliers are indicated. *P*<0.05 was considered statistically significant.

## Supplementary Material

Supplementary information
