# Supplementary material for: Rho kinase-dependent apical constriction counteracts M-phase apical expansion to enable mouse neural tube closure
Source: J Cell Sci. 2019 Jul 1;132(13):jcs230300. doi: 10.1242/jcs.230300 (PMC6633395; doi:10.1242/jcs.230300)
Supplement: Supplementary information [file joces-132-230300-s1.pdf]

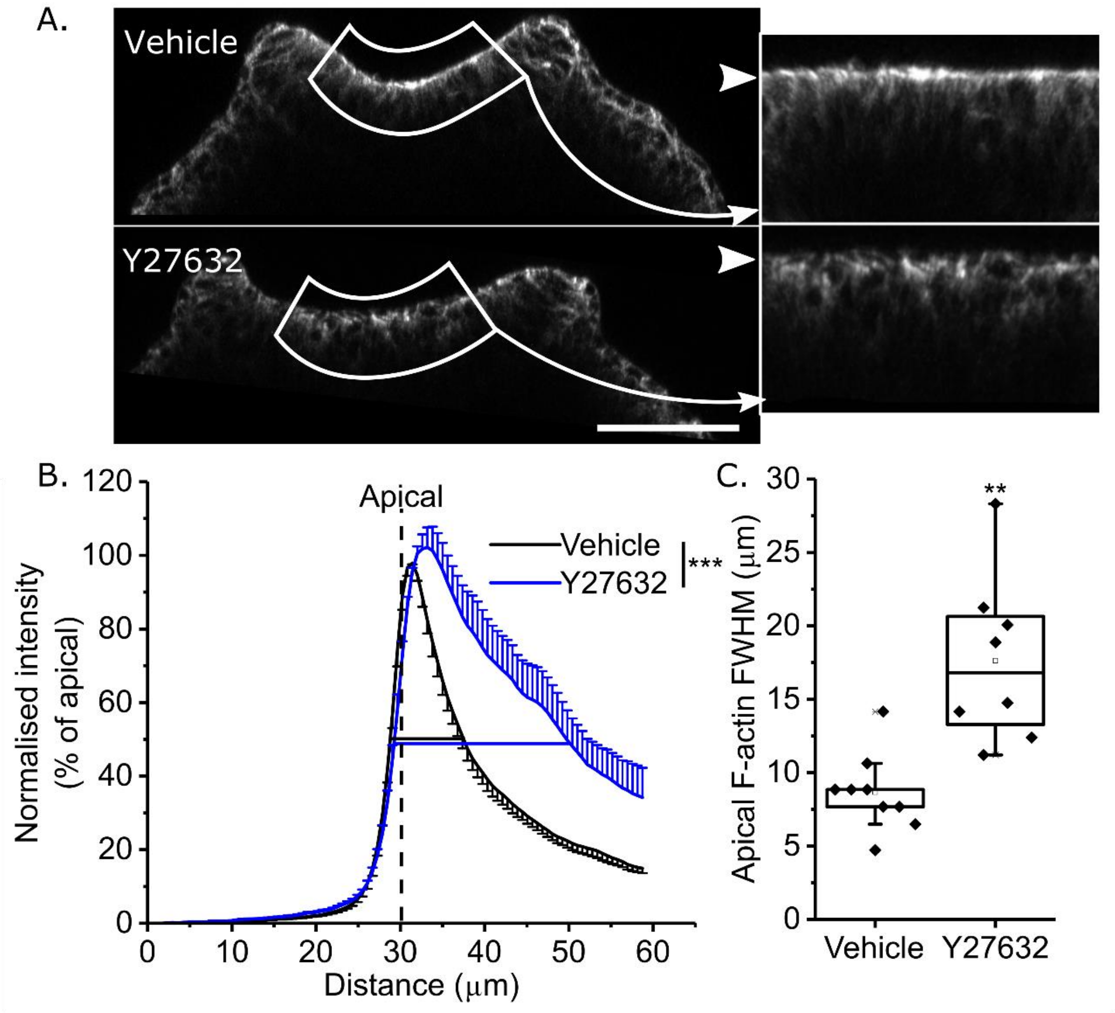

Figure S1: Rock inhibition diminishes F-actin apical localisation. A) Optically-resliced cross-sections through wholemount phalloidin-stained vehicle and 10 µM Y27632-treated embryo PNPs after 8 hours of treatment. The curved apical surfaces are straightened in the inset (apical surface indicated by the arrowhead). Scale bar = 100 µm, grey LUT. **B)** Line profile of F-actin intensity centred on the apical surface (dashed line). The profiles shown were measured at 75% of the PNP's length from the rostral end and are shown as the average of n = 9 vehicle and n = 8 10 µM Y27632-treated embryo PNPs. Each profile was corrected for background noise by subtracting the background signal 30 µm above the apical surface and the apical surface was set at 100% in each embryo. The horizontal lines indicate the approximate full-width half-max (FWHM) values which indicate the thickness of the apical enrichment. **C)** Comparison of neuroepithelial FWHM apical F-actin thicknesses between vehicle and 10 µM Y27632-treated embryos, showing more selective enrichment to a narrow apical domain in vehicle-treated embryos versus more diffuse staining in Y27632-treated embryos.

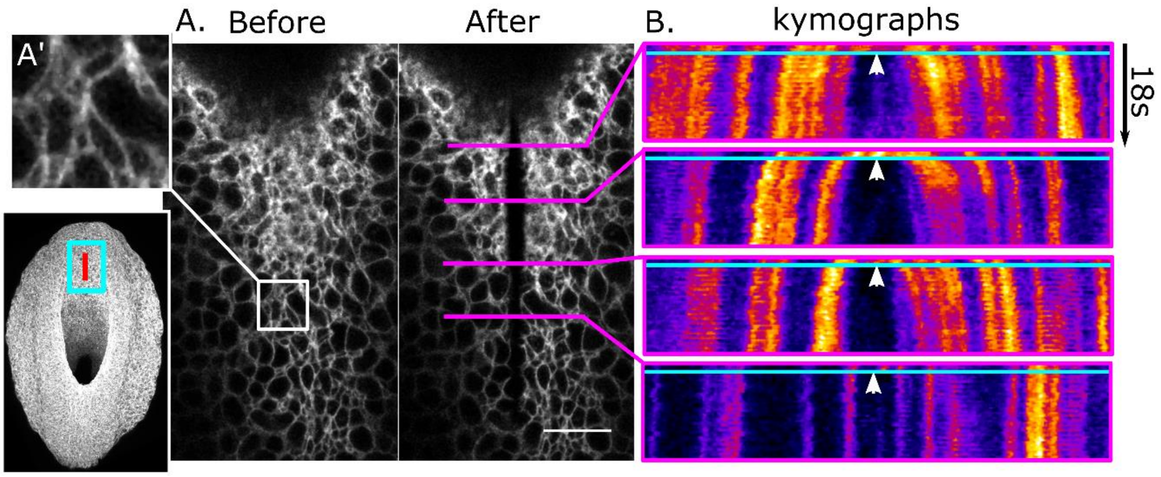

Figure S2: Linear ablations in the open region of the PNP produce excessively variable recoil. A) Optical section through a live-imaged CellMask-stained PNP before and after a linear laser ablation (red line in the representative whole-PNP view with the imaged region shown by the cyan box). Scale bar = 25 µm. The cell borders in the pseudostratified epithelium are highly variable (magnified view in **A'**) and most cannot be selectively ablated. **B)** Representative kymographs at the indicated positions along the linear ablation in **A** showing highly variable recoil within the same ablation. The white arrow indicates the centre of the ablation.

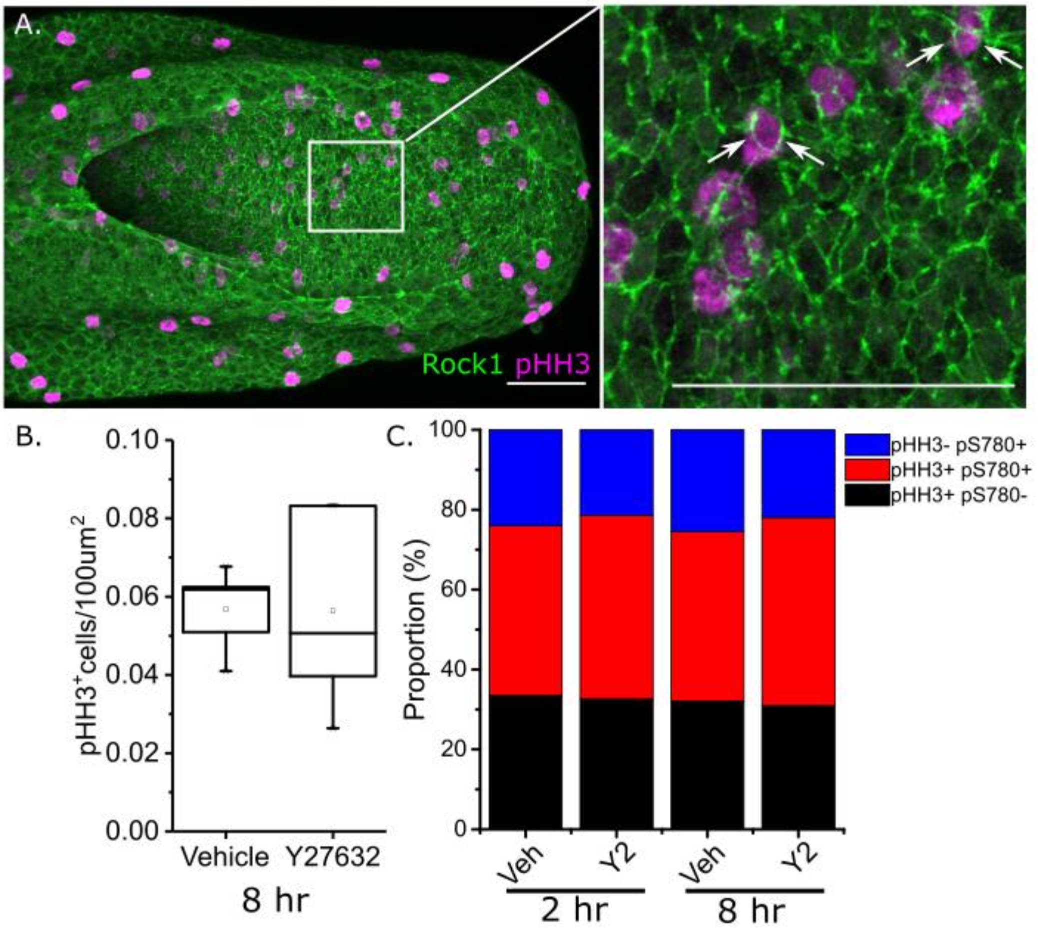

Figure S3: Rock inhibition does not alter neuroepithelial cell mitotic index. A) Rock1 is enriched around the apical borders of neuroepithelial cells, including cells which are pHH3 positive (arrows). Scale bars = 50 µm. **B)** Quantification of the number of pHH3 positive cells per 100 µm^2 of PNP in vehicle (n = 5) and 10 µM Y27632-treated (n = 7) embryos after 8 hours of culture. **C)** The proportion of neuroepithelial cells labelled singly with pHH3 or pS780, or double-labelled with pHH3 and pS780, as a proportion of cells labelled with either after 2 hours (vehicle = 230 cells from 6 embryos, Y27632 = 196 cells from 5 embryos, X^2 p = 0.71) or 8 hours (vehicle = 224 cells from 5 embryos, Y27632 = 378 cells from 8 embryos, X^2 p = 0.48).

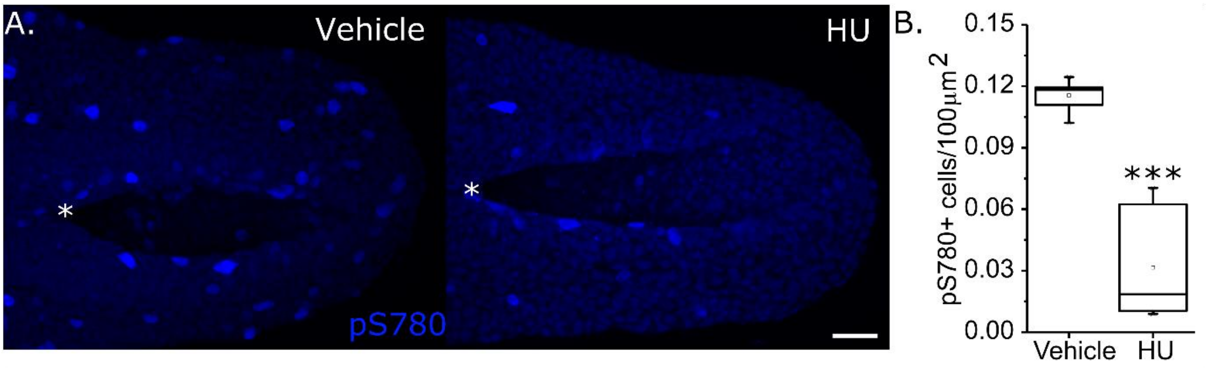

Figure S4: HU treatment reduces neuroepithelial mitotic index. A) Representative wholemount maximum projections showing the PNPs of vehicle and 0.8 mM HU treated embryos after 8 hours of culture. * indicates the zippering point, scale bar = 100 µm. B) Quantification of the number of pS780+ cells per 100 μm^2 of PNP in vehicle 0.8 mM HU treated embryos after 8 hours of culture. N = 6 per group, *** $p < 0.001$.

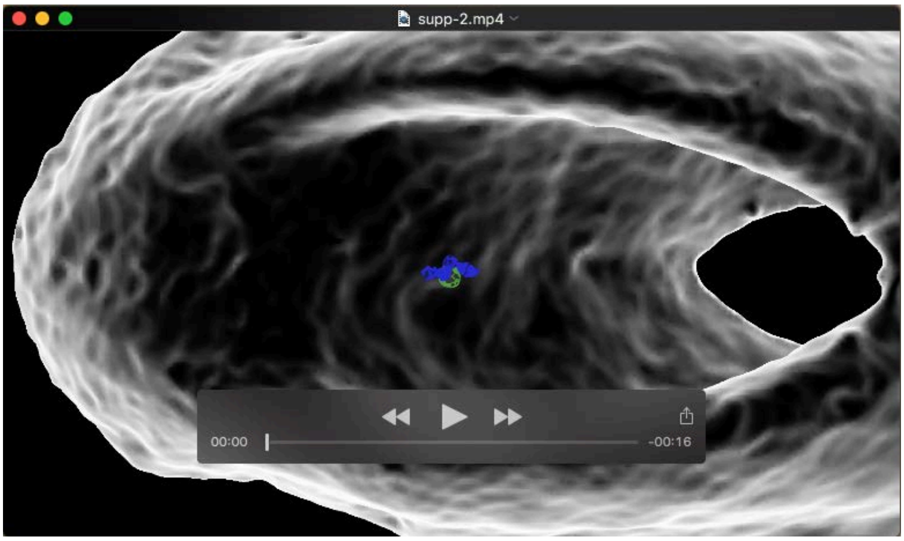

Movie 1: 3D illustration of a pHH3+ (green) neuroepithelial cell surrounded by three non-mitotic (blue) neuighour cells. Note that the pHH3+ cell has a small apical surface relative to its neighbours, which wrap over its widest part.
